# Building equitable foundations: policy solutions to address preschool suspension, expulsion, and exclusion inequities

**DOI:** 10.3389/fpsyg.2026.1726081

**Published:** 2026-05-19

**Authors:** Nina Smith, Valerie Jarvis McMillan, Sherrell Hicklen House, Jennifer Beasley, Jessica Price

**Affiliations:** 1 College of Health and Sciences, North Carolina Central University, Durham, NC, United States; 2 Department of Family and Consumer Sciences, North Carolina Agricultural and Technical State University, Greensboro, NC, United States; 3 Center for Youth, Family and Community Partnerships, University of North Carolina at Greensboro, Greensboro, NC, United States; 4 Childcare Network Inc., Raleigh, NC, United States

**Keywords:** early childhood, exclusionary practices, expulsions, policy, suspensions

## Abstract

This work contributes to the recent special issue in Frontiers in Psychology, titled “Bridging Racial, Economic, and Intersectional Equity Gaps in Developmental and Educational Psychology: The Key Roles of Caregivers and Early Childhood Interventions and Approaches,” which calls attention to the important role of innovative approaches to protect and nurture the learning experience of young children. The present conceptual review paper aims to provide policy recommendations to education researchers, practitioners, instructors, and other key stakeholders to bring awareness to and eradicate the practice of suspension, expulsion and exclusionary practices in early care and learning environments. Accordingly, by drawing on Critical Race Theory, we highlight the positive efforts of the *North Carolina Coalition for Inclusion, Not Expulsions* as a model for paving the way toward the elimination of harsh disciplinary methods used in preschool contexts. Then, we introduce several policies and objectives that should be enacted across all systems that govern preschool settings. Finally, we conclude with thoughtful implications and future directions.

## Introduction

All young children, regardless of their background, deserve the strongest possible start. That start includes access to high-quality early care and education. For young children, ages two to five, early care and educational experiences create a promising early foundation for success in school, career and life (Bustamante et al., 2023). Yet, year after year, data shows that Black preschoolers are significantly more likely to be suspended or expelled than White preschoolers (Iruka, 2019; Zinsser et al., 2022). This means an unexpected journey for Black children, in which they are far too likely to miss out on early childhood experiences that help unlock their fullest potential by supporting healthy brain development during their critical early years.

The most recent available data shows that Black preschool children represent about 18 percent of total preschoolers but make up more than 40 percent of suspended preschoolers (Office of Civil Rights Data Collection, 2018). The impact of early care and education suspensions also extends well beyond preschool doors. Pushing young children out of early care and education settings has broad and long-term consequences for children, families, and our economy. Families deserve to know that their children are safe, welcomed, and cared for in early care and education settings while they work. Data consistently shows that these unfair disciplinary practices put young children on track toward more suspensions, lower overall academic achievement and higher dropout rates that even lead to higher rates of incarceration (Allen et al., 2022; Iruka, 2019). When young children get removed from care, parents and caregivers are forced to take time off work or get pushed out of the workforce entirely (Gilliam et al., 2016). This hurts caregivers’ ability to support their families and creates instability in businesses across the economy. And, without better preparation and resources like mental health consultation support, the early childhood workforce lacks access to critical tools needed to provide positive environments for all young learners to grow and develop.

These disciplinary practices often reflect implicit biases and contribute to the preschool-to-prison pipeline by setting a precedent for repeated, unnecessary removal. Suspending and expelling preschoolers results in significant policy implications, particularly in terms of equity, child development, and long-term educational outcomes (Al-Shamma et al., 2018). Policymakers must address these disparities by implementing policies and practices that promote inclusive, trauma-informed approaches in early education settings. This includes professional development for educators, clear guidance on supporting children including behavioral interventions, and limiting suspension and expulsion as a solution for intervention.

Furthermore, removing young children from educational environments disrupts their critical early learning experiences and can hinder social–emotional development. From a policy perspective, this not only undermines investments in early childhood education but also increases the likelihood of future academic struggles, behavioral issues, and lifelong challenges. States and school systems have taken note and are increasingly responding by enacting legislation that restricts or bans the use of suspension and expulsion in preschool settings, instead encouraging the use of restorative practices and mental health supports. These policies aim to ensure all children receive equitable access to quality early learning opportunities, laying a stronger foundation for lifelong success.

Guided by a Critical Race Theory (CRT) perspective, this paper describes a solutions-based movement designed to address a collaborative group of dedicated partners committed to being a part of the movement to eliminate racial inequities in suspensions, expulsions, and exclusions in early care and education settings. The movement seeks to unearth the unexpected journey of young Black children and their families that experience suspension, expulsion, and exclusionary practices in early care and education settings, describe a solutions-based movement designed to address and eliminate the unexpected journey, and elucidate policy recommendations that have resulted from the work of the *North Carolina Coalition for Inclusion, Not Expulsions*. Centralizing many of the CRT tenets in our work (Delgado and Stefancic, 2023) (e.g., race and racism, intersectionality, voices of color, and critique of colorblind ideologies), we developed this movement with these tenets at the forefront by taking an antiracist stance to advance significant transformation concerning early care and education suspension, expulsion, and exclusionary practices for Black children in North Carolina. The work of the *North Carolina Coalition for Inclusion, Not Expulsions* can be used as a model for other states tackling this important issue. Broadly, our goal is to highlight two priority areas for policy makers and ECE stakeholders: (1) ensuring that ECE professionals are equipped with the resources and relational tools necessary to build affirming partnerships with families and their children (2) addressing the root causes of racism and discrimination that underlie exclusionary practices.

## Critical race theory

Grounded in CRT, our movement advocates for understanding and addressing the impact that systemic racism can have on our young children, and importantly, moving toward a systemic solution/transformation. This movement seeks to illuminate the unjust experiences of young Black children and their families who encounter suspension, expulsion, and other exclusionary practices in early childhood educational contexts using a CRT lens. CRT provides a powerful lens through which to examine how systemic racism is embedded in educational policies and practices (Delgado and Stefancic, 2023). Central to CRT is the acknowledgement that racism is not an isolated occurrence but a normalized and enduring feature of American society, which includes early care and educational practices.

This movement centers on several foundational tenets of CRT. First, it acknowledges that race and racism are socially constructed and deeply embedded in the structures of early childhood education (ECE), influencing how policies are developed and implemented. For example, disciplinary policies that appear neutral on the surface often disproportionately impact Black children due to implicit biases and cultural misunderstandings, reinforcing systemic inequities (Allen et al., 2021; Gilliam et al., 2016; Love and Beneke, 2021).

Second, the principle of intersectionality informs our understanding of how race interacts with other identity markers such as socioeconomic status, gender, and ability to shape the lived experiences of Black children (particularly Black boys) and their families in complex and compounding ways. For instance, Black boys with disabilities are more likely to be labeled as disruptive and are at greater risk for exclusionary discipline, reflecting the compounded effects of racism and ableism (Annamma et al., 2013; Love and Beneke, 2021).

Third, the voice of color tenet is operationalized through the intentional inclusion of narratives from families, educators, and community members of color. These counter stories challenge dominant narratives that often obscure or minimize the impact of racial bias in disciplinary practices. For example, the lived experience of Black parents reveals patterns of surveillance, deficit-based assumptions, and exclusions that are not captured in quantitative data alone, offering a more nuanced understanding of systemic harm (Delgado and Stefancic, 2023). Fourth, our work critiques colorblind ideologies that claim to treat all children equally while ignoring the historical and structural inequities that disproportionately affect Black children. Early childhood institutions often reinforce white, middle-class norms of behavior and development, leading to the marginalization of children who do not conform to these standards (Allen et al., 2021; Gilliam et al., 2016; Love and Beneke, 2021).

The *North Carolina Coalition for Inclusion, Not Expulsions*, embodies these tenets through its collaborative, community-driven approach. By taking an explicitly antiracist stance, the Coalition works to dismantle exclusionary practices and promote equity in early childhood settings. The Coalition’s efforts include elevating community voices, advocating for policy change, and developing practical tools and resources for ECE professionals. These actions reflect CRT’s commitment to praxis, linking theory with action to achieve social justice.

This movement not only documents the unexpected and often traumatic journeys of Black children and their families through exclusionary ECE systems but also highlights a strengths-based movement aimed at systemic transformation. The Coalition’s work offers a replicable model for other states seeking to address racial inequities in early childhood discipline. Specifically, we emphasize two critical policy priorities for stakeholders and decision-makers: (1) ensuring that ECE professionals are equipped with the resources and relational tools necessary to build affirming partnerships with families and (2) addressing the root causes of racism and discrimination that underlie exclusionary practices. Through this CRT informed lens, the movement contributes to a growing body of work committed to equity, justice, and the reimagining of early childhood education.

## National landscape

The kindergarten through twelfth grade (K-12) setting has long received attention for suspension and expulsion practices, with many scholars noting disparate patterns among minority youth (Leung-Gagné et al., 2022). Of key concern are the increased rates of these practices in early care and education settings. One of the first large scale studies found that expulsion rates for preschool children were already markedly higher than for K-12 students (Gilliam, 2005; Zeng et al., 2019). For every 1,000 preschoolers, roughly 6.7 were expelled, compared to 2.1 per 1,000 K-12 students. The past few decades have witnessed persistently high rates of preschool suspensions and expulsions. There have been positive movements, such as increased attention, policy changes, and support. However, this traction has not been consistent, nor has it been uniform across states. For example, a recent study by Giordano et al. (2025) revealed that reported suspension rates were lower in 2018 but then increased significantly by 2022. The effects remained through 2024. The Covid-19 pandemic drove decreases in both suspension and expulsion rates. Yet, post-pandemic rates are similar to trends prior to 2020. According to the National Association for the Education of Young Children (NAEYC), almost 9,000 three- and four-year-old children are expelled from their state-funded early learning environment (Stegelin, 2018). Most alarming are the gendered and racial/ethnic disparities across reports of suspensions, expulsions and exclusionary practices. Disproportionately, Black children experience disruptions in their early learning opportunities more than their White peers—with Black boys experiencing the highest rates. Black children represent 19% of preschool enrollment, yet they comprise 47% of the share of preschool children receiving one or more out of school suspensions (Office of Civil Rights Data Collection, 2018).

The disparate rates of suspension, expulsion, and exclusionary practices are likely due to inconsistencies across states. There are several definitions for the practice of suspension and expulsion, and they vary greatly by state. Some states have ranges from 3 to 20 days, while others indicate the rest of the semester. Each state and/or school system is also bound to local discretion. At the preschool level, there are fewer formal definitions or parameters, and some states disallow expulsion. According to the Education Commission of the States (ECS), states are increasingly addressing this issue through state level legislation (Novoa and Malik, 2018). A handful of United States’ states have emerged as frontrunners in the elimination of suspension and expulsions of young children. Moreover, these efforts have even extended protections through kindergarten and early elementary grades. There are exceptions for serious misconduct, such as weapons and extreme violence, and emphasize the importance of mental health resources and other preventive supports. Specifically, California, Connecticut and Maryland stand out as exemplary states that have moved toward stricter limits on harsh discipline practices in early learning settings. Among the first states to prohibit out-of-school suspension and expulsion for preschool children was Connecticut under the establishment of Public Act SB 1053. Similarly, Maryland has implemented significant legal safeguards for young children. In 2017, the state passed statutes banning suspension and expulsion of children in public prekindergarten through second grade classrooms. When suspensions are permitted, due to extreme cases, they are limited to no more than 5 days, and the school administration is required to consult with a mental health professional. State-funded preschools in California are required to document attempts to facilitate resolutions to challenging behaviors before employing expulsion. Recent laws have been passed that forbid suspension and expulsion in child care programs for infants and toddlers. Of note, there are important procedural caveats within each state that should be considered. The thresholds for exception vary such that what counts as violent or serious harm are subject to interpretation. Therefore, what ECE educators and staff determine as warranting suspension and/or expulsion is likely subject to interpretation and could influence how strictly or loosely the rules are applied. Correspondingly, the lack of universality of laws across states allows ECE settings to determine how rules are implemented. For example, the state of California’s laws apply to child care programs that fall under the *California State Preschool Program* or family or home childcare networks. Providers that fall outside of these networks may have different rules (e.g., programs governed by public school systems, charter/magnet schools, or private preschools). Critical to eradicating harsh disciplinary practices are the implementation of provisions that involve parents, mental health resources, and screening measures.

There are several other states that are making positive strides toward abolishing suspension, expulsions, and exclusionary practices in ECE contexts. Arkansas, Colorado, Ohio, and Tennessee continue to make significant investments in ECE mental health consultation and other preventive supports. Although these states have not enacted full bans, this is a promising start that can yield enormous outcomes. Oregon is another state that is revamping its ECE landscape to be more inclusive (Mendoza, 2023). The state’s Department of Early Learning and Care (DELC) recently required the legislation of a ban on suspensions and expulsions in DELC-funded programs effective July 1, 2026. At the same time, Oregon’s *Early Childhood Suspension and Expulsion Prevention Program* (ECSEPP) was created to build systems infrastructures such as technical assistance, training and racial equity/trauma informed ideologies to aid ECE staff in managing challenging behaviors without employing exclusionary practices.

There are still states lagging with mean expulsion rates above the national average. Examples include Alabama, Florida, Georgia, Kentucky, North Carolina, South Carolina, Texas, and Virginia. Although among this group, several efforts are underway in North Carolina to eliminate suspension, expulsion and exclusionary practices in ECE settings. That work is highlighted in the section to follow.

## The North Carolina coalition for inclusion, not expulsions: past, present, future

The *North Carolina Coalition for Inclusion, Not Expulsions* (a.k.a. Coalition) is a collaborative group of dedicated partners who have a passion for building a movement toward addressing and eliminating suspensions, expulsions and exclusionary practices in early care and education settings. The Coalition includes researchers, early educators, directors of centers, administrators, specialists, families, and advocates with distinct expertise in and experiences across the early childhood field. Together, the members of the Coalition are collectively working to envision a North Carolina where the youngest Black children—ages two to five—and their families have fair and equitable opportunities and experiences to fully participate in all early care and education settings. The Coalition is on a bold mission to build a *movement* that transforms early care and education to:

- Better meet the needs of North Carolina’s diverse children and families.
- Strengthen our state’s essential early childhood workforce.
- Create a model for other states.

In 2022, the Coalition developed a strategic plan that included a logic model consisting of resources, activities and interventions, as well as processes to measure and short- and.

long-term outcomes aimed at the following: (1) eliminating racial inequities in early care and education suspensions, expulsions and exclusionary practices; (2) dramatically reducing the overall number of suspensions and expulsions; and (3) providing alternative tools for early care and educational professionals and for families to offer positive responses to children’s behaviors. In 2023, the Coalition used its logic model has a basis to inform its 10-year goals and initial action steps, including guidance the following: ongoing feedback from Coalition members; insight from dozens of experts across North Carolina and the country; a series of community listening sessions with early care and education professionals and parents, caregivers and families conducted across eight North Carolina counties; and a state and national research analysis.

As with any strategic plan, it is noted that the plan is a living document that will evolve over time. But the Coalition will remain focused on its overarching vision: to ensure Black children and families have an equitable opportunity to participate in early care and education in North Carolina.

### How do we get there?

The Coalition’s work is actualized through five Task Forces with specific focus areas. These Task Forces focus on communications, research and data, public policy and systems infrastructure; program and practice evaluation; resources and support, and communications. The section below provides a brief description of each Task Force.

### How we do the work

To identify the most promising solutions, the Coalition includes five Task Forces, each with a specific focus area.

#### Communications Task Force

The Communications Task Force provides feedback, communications support and community engagement expertise for efforts to build public awareness of this issue.

#### Research and Data Task Force

The Research and Data Task Force assess available North Carolina and national research and data, identifies any important gaps in data and best practices for capturing, and provides reports on that data.

#### Program and Practice Evaluation Task Force

The Program and Practice Evaluation Task Force seek to understand existing successful programs and strategies in North Carolina and across the country, as well as identify and shared learnings that have specific innovative approaches and practices that are safe, culturally sensitive, relevant, and sustainable in early care and education settings.

The Task Force is developing a definition of a *Promising Model* that is specifically aimed at addressing and eliminating suspensions, expulsions, and exclusions in early care and education, especially among young Black children.

#### Resources and Support Task Force

The Resources and Support Task Force provide reports, articles, books, websites, etc. across the state and nationwide serving as resources to support opportunities to create, test and/or evaluate promising practices and approaches for addressing and eliminating suspension, expulsion and exclusionary practices in early care and education.

#### Public Policy and Systems Infrastructure Task Force

The Public Policy and Systems Infrastructure Task Force is exploring and recommending the most effective policy solutions that will end the practice of removing children from early care and education settings.

To realize the vision and mission, the Coalition is pursuing bold policy and practice solutions that will lead to the following outcomes:

- By 2027, we will identify and share innovative, actionable, replicable solutions and tools to eliminate racial inequities in preschool suspensions, expulsions, and exclusionary practices.
- By 2029, North Carolina has taken steps to eliminate early care and education suspensions, expulsions, and exclusionary practices across the state.
- By 2034, North Carolina has equitable opportunities for Black children to participate in early care and education settings.

Core to the mission of the Coalition is a transformative public policy that leads to changes that North Carolina communities can see, hear, and feel. Recommending the most effective policy solutions that will end the practice of removing children from early care and education settings is a top priority of the Coalition’s movement toward addressing and eliminating suspension, expulsion, and exclusionary practices disproportionately experienced by Black children and their families.

## Policy priorities

Policies are vital to the elimination of suspensions, expulsions, and exclusionary practices in ECE settings. They not only establish a framework that is systemic in nature but also signal a commitment to interrogating the cause of exclusionary practices. Effective policies set clear and consistent limits and help curate environments where challenging child behavior is met with skillful intervention rather than punitive methods. Informed by the work of the North Carolina Coalition on Inclusion, Not Expulsion as well as the work of frontrunner states leading the movement, there are key policy levers that should be considered as the movement toward eliminating harsh disciplinary practices across the ECE landscape.

### Ensuring ECE professionals have the resources and tools to build positive relationships with families

#### Objective 1.A

Advocate for policymakers to allocate funding for a tailored assistance program (e.g., S/E PD, IECMH consultation) within the Division of Child Development and Early Education to support the elimination of suspensions and expulsions.

To improve social–emotional development and reduce the use of exclusionary discipline in early childhood contexts, funding should be dedicated to the development and implementation of targeted assistance programs. These programs would provide professional development (PD) in social–emotional (S/E) learning and access to Infant and Early Childhood Mental Health (IECMH) consultation services. Tailored coaching and reflective supervision should be embedded to support early educators in understanding and responding to children’s behavior in developmentally appropriate and trauma-informed ways. These programs should prioritize equity, recognizing the disproportionate rate at which Black children are suspended, expelled, or excluded.

#### Objective 1.B

Advocate for policymakers to reduce the statewide staff-to-child ratio (i.e.: an administrative policy).

High staff-to-child ratios can impact the ability of educators to form strong, individualized relationships with children and their families. Reducing these ratios would allow for more responsive caregiving, better communication with families, and more opportunities to observe and address children’s needs. Policy should aim to meet or exceed national recommendations, especially in classrooms serving infants and toddlers, where close, nurturing relationships are foundational to development.

#### Objective 1.C

Advocate for improved family engagement resources for all caregivers.

Policies should ensure that educators and programs have access to high-quality, culturally responsive, and sustainable family engagement materials. This includes multilingual communication tools, family engagement toolkits, and training in home-visiting models. Family engagement must be highlighted as a shared responsibility that respects, appreciates, and uplifts family’s voice and choice and emphasizes the importance of families as partners in a child’s development, especially in Black communities.

#### Objective 1.D

Advocate for a centralized access point/support line for administrators to utilize when support is needed.

Create a state-level or regional support line where early learning administrators can quickly access expert consultation, technical assistance, mental health resources, and behavioral support. This centralized system should be staffed by professionals with expertise in early childhood development, lived experience, trauma-informed care, inclusion, and culturally responsive practices. This would provide timely intervention, reduce educator stress, and support the care for children demonstrating challenging behaviors.

### Addressing the root causes of racism/discrimination

#### Objective 2.A

Advocate for professional development models that explicitly address culturally responsive practices.

Continuous professional development (PD) must include comprehensive training in culturally responsive teaching, anti-bias education, and the impact of systemic racism on child development. These PD models should be developed with input from diverse communities having lived experience and include components such as self-reflection, implicit bias training, and strategies to create inclusive classroom environments. PD should also include mentorship and accountability measures to ensure that it is research informed and consistent with current changes.

#### Objective 2.B

Establish funding for programs that want to replicate proven successful models that show evidence of narrowing racial disparities.

Advocate for state and federal funding streams to support the replication and scaling of programs with strong data demonstrating reduced disparities in discipline, access, and developmental outcomes among Black children. This includes programs that employ community-based, culturally rooted practices; family engagement strategies with proven effectiveness in marginalized communities; and partnerships with historically Black, Indigenous, and people of color (BIPOC)-led organizations.

#### Objective 2.C

Provide program administrators with guidance and tools that empower them to support students in the classroom.

Early childhood program leaders should have access to toolkits, coaching, and technical assistance focused on implementing equitable policies and supporting inclusive teaching practices. These tools should help administrators assess classroom environments, implement trauma-informed and anti-racist approaches, and mentor their staff using culturally relevant and sustainable practices. Policies should incentivize leadership development opportunities for administrators from underrepresented backgrounds and include accountability measures to track progress toward equity goals.

We know that creating equitable opportunities for Black children to participate in early care and education contexts will take time, collaboration and intentional, strategic action through policy and practice. The future of disciplinary practice in early childhood education depends on intentional investment in equitable, developmentally grounded systems of support. The recommendations outlined here emphasize the importance of equipping professionals with the tools to build strong, trust-based relationships with families, eliminate exclusionary discipline, and address structural and systemic contributors to inequity. Culturally responsive professional development, adequate staffing, accessible mental health consultation, and centralized administrator support are foundational in fostering positive child outcomes.

To move this work forward, the next critical step is coordinating advocacy for policy change at the state level, beginning with targeted funding and administrative reform. These changes must be informed by both developmental science and lived experiences, while centering on the voices of families. When systems prioritize an expected journey of inclusion, connection, and equity, they create environments where Black children and their families can grow and thrive.

## Implications and future directions

This movement, grounded in CRT, represents a movement for change. It seeks to disrupt and transform the systems that perpetuate racial inequities in early care and education, particularly those that result in the suspension, expulsion, and exclusion of Black children. By centering race and racism as foundational elements to dismantle, the movement challenges the assumption that exclusionary discipline practices are neutral or objective. Instead, it reveals how these practices are shaped by historical patterns of marginalization. This movement calls for a shift from reactive disciplinary reforms to proactive, equity-centered strategies that are rooted in justice and community voice.

A central implication of this work is the need to equip early care and education professionals with relational tools that foster affirming partnerships with families. These tools include culturally responsive communication strategies, reflective supervision, and trauma-informed care practices. For example, educators can engage in home visits to build trust, use family-teacher conferences that prioritize listening and shared decision-making to increase family engagement, and implement two-way communication systems that respect linguistic and cultural diversity (National Association for the Education of Young Children, 2021). These approaches help educators move from deficit-based assumptions to strength-based relationships that honor family expertise and promote mutual respect.

Equally important is the imperative to address the root causes of racism and discrimination that underlie exclusionary practices. This involves examining and dismantling structural inequities such as biased assessment tools, funding disparities, and the dominance of white, middle-class norms in early childhood curricula. Strategies to confront these root causes include revising discipline policies to incorporate restorative practices and embedding anti-racist education into daily curriculum and interactions. Anti-racist teaching requires educators to name and challenge racism when it occurs, support children in processing inequities, and create inclusive environments that affirm diverse identities (Allen et al., 2021). Leadership structures must also reflect the racial and cultural diversity of the communities served, and policies must be scrutinized for implicit bias and exclusionary effects.

It emphasizes two critical policy priorities for stakeholders and decision-makers. First, early care and education professionals must be equipped with the resources and relational tools necessary to build affirming partnerships with families. Second, systemic efforts must be directed toward addressing the root causes of racism and discrimination that drive exclusionary practices. These priorities are not only essential for reducing disparities but also for transforming early childhood education into a safe space for Black children and families.

Future directions for this movement include expanding CRT informed interventions across diverse early childhood contexts, engaging in participatory research that centers family and community voices, and advocating for sustained policy change. Despite ongoing debates surrounding diversity, equity, and inclusion initiatives, the coalition’s approach can serve as a model for other states seeking to reduce suspensions, expulsions, and other exclusionary practices. Globally, there rests an opportunity to expand our reach and model efforts internationally. Many countries have witnessed a vast increase in immigration from African, Asian, and Latin American populations. Colonial systems have historically imposed Eurocentric standards of language, behavior, and knowledge, positioning them as superior while devaluing Indigenous and local ways of knowing. Similarly, caste and apartheid systems institutionalized rigid social stratification, normalizing differential expectations for children based on identity. These legacies can still surface in early childhood settings through implicit bias, lowered expectations for marginalized children, disproportionate discipline, and the privileging of certain cultural expressions over others. As a result, some children are more likely to be viewed through deficit-based lenses rather than as capable learners with diverse strengths. Even in an international setting, contemporary educators can recognize and disrupt these inherited patterns. This involves critically examining how historical inequities shape classroom practices, curricula, and relationships with families, and intentionally adopting approaches that affirm children’s identities and lived experiences. By acknowledging the lasting impacts of colonization, caste, and apartheid, global educators can move toward more just and humanizing practices that support all children’s development and sense of belonging. Longitudinal efforts can help assess the impact of inclusive, antiracist practices on child development and educational outcomes. Ultimately, this movement contributes to a growing collective effort to reimagine early childhood education as a system that affirms the dignity and potential of every child.
